# Relationship between sleep quality and dietary nutrients in rural elderly individuals: a latent class analysis

**DOI:** 10.3389/fnut.2025.1479614

**Published:** 2025-01-20

**Authors:** Xinlei Zhao, Xinyan Xie, Han Zhou, Feng Zhong, Cuiping Liu

**Affiliations:** ^1^School of Public Health, Qingdao University, Qingdao, China; ^2^Shandong Center for Disease Control and Prevention, Qingdao, China; ^3^School of Nursing, Shandong First Medical University & Shandong Academy of Medical Sciences, Taian, China

**Keywords:** rural elderly, sleep pattern, latent class analysis, dietary nutrition, influence factor

## Abstract

**Background:**

This study sought to identify sleep patterns in older adults residing in rural regions, as well as investigate the potential influence of dietary nutrient intake on these patterns.

**Methods:**

Data were collected from a cross-sectional sample of Qingdao Town, Shandong Province, China. The study investigated 1,167 elderly participants using a general questionnaire, the Pittsburgh Sleep Quality Index, the simplified Food Frequency Questionnaire, and 24-h dietary recall methods. Latent profile analysis and binary logistic regression were applied for data analysis.

**Results:**

Two sleep categories were identified as “Quick falling asleep, long time, high efficiency group,” Class 1(89.1%) and “Difficult falling asleep, short time, low efficiency group,” Class 2(10.9%). In comparison to Class 1, individuals in Class 2 exhibited a higher likelihood of experiencing difficulties in falling asleep quickly and having poor sleep efficiency when consuming less than 50 g/d of potatoes (OR = 1.863, *p* = 0.034). Conversely, a daily protein intake of 60 g or more (OR = 0.367, *p* = 0.007), a daily intake of retinol of 700 equivalents or more (OR = 0.212, *p* = 0.002), and a daily milk intake of 300 g or more (OR = 0.295, *p* = 0.035) were associated with a greater probability of falling asleep quickly, having longer sleep duration, and experiencing higher sleep efficiency.

**Conclusion:**

Our analysis identified two distinct sleep quality patterns among elderly individuals in rural areas. The sleep quality of rural elderly individuals is influenced by their dietary habits. The findings demonstrated a positive association between enhanced sleep quality and higher intake of dairy products, potatoes, and foods containing retinol and protein. Therefore, we propose increased consumption of these nutritional sources for the elderly population.

## 1 Introduction

The quality of sleep among the elderly is a growing concern in terms of public health. Sleep disorder has a significantly negative impact on morbidity and mortality, particularly in the elderly population (1–4). In China, which has the largest elderly population worldwide, the aging populace has led to a substantial health burden (5). Mild sleep disturbances can lead to fatigue, irritability, daytime dysfunction and slowed responses (6). Chronic sleep problems can lead to an increased risk of mortality and contributes to both the individual risk and societal burden associated with several medical epidemics, including cardiovascular disease, diabetes, obesity, and cancer (7). A discernible variance was observed in various facets and health concerns pertaining to health literacy among urban and rural demographics, with a lower health literacy level noted among rural individuals with limited educational attainment (8). Elderly individuals in rural areas, due to limited access to healthcare services and lack of knowledge, tend to refrain from seeking assistance for sleep problems, rendering them a high-risk demographic frequently neglected (9).

The evaluation of sleep quality in elderly individuals in most studies is conducted using the Pittsburgh Sleep Quality Index (PSQI) index sorely. Given the intricate nature of sleep issues in the elderly, conventional analysis methods of PSQI may not accurately assess individual sleep quality. In healthy older adults, the PSQI is influenced by both the total duration of sleep and the cognitive ability of older individuals to recall experiences from the past month (10). Emphasizing the total score alone might disregard the associations between individual questionnaire items and sleep quality. Latent class analysis (LCA), a probabilistic modeling technique, enables data clustering and statistical inference (11). Particularly applicable to data featuring categorical variables, LCA focuses on individual variances within latent classes rather than their magnitudes. LCA offers advantages over traditional clustering methods by providing fit statistics and enabling the incorporation of covariates in models (12). Consequently, LCA emerges as a more suitable approach for analyzing sleep issues in the elderly and accurately categorizing their sleep quality. Notably, there is a dearth of research utilizing LCA to classify the sleep quality of the elderly, underscoring the potential of this study to inform future research endeavors.

Dietary nutrition is believed to significantly affect sleep quality, with different nutritional supplements being used to enhance sleep. Nutrition can profoundly influence hormone levels and inflammation status, both of which directly or indirectly contribute to the onset of insomnia (13). Due to differences in economic conditions and dietary habits, rural elderly populations are more susceptible to nutrition-related issues. Previous researches on the relationship between nutrition or nutrients and sleep have focused on the association between specific foods, nutrients, nutritional components (14), or certain dietary patterns (15, 16) and overall sleep quality (17). Studies have emphasized that adhering to a healthy diet is associated with a lower prevalence of insomnia symptoms, while sticking to unhealthy patterns is linked to an increased prevalence of insomnia (18). The Mediterranean diet pattern can improve the sleep quality of the elderly, as diet may influence sleep through melatonin and its biosynthesis from tryptophan. Experimental data suggests that providing specific foods rich in tryptophan or melatonin can improve sleep quality. Diets rich in fruits, vegetables, legumes, and other sources of tryptophan and melatonin have been shown to predict good sleep outcomes (19). There is also evidence linking pro-inflammatory diets to daytime dysfunction, sleep onset awakenings, and sleep apnea (20). In conclusion, the relationship between nutritional components and sleep is complex, given the variability in dietary patterns and the reliance on individual digestive and metabolic processes. Thus, further investigation is warranted to elucidate the connection between dietary nutrition and sleep patterns in elderly individuals residing in rural areas.

Hence, the objective of this study is to employ LCA to uncover latent sleep patterns among rural elderly individuals and examine the relationship between dietary nutrients and diverse sleep patterns.

## 2 Methods

### 2.1 Design and data collection

This study included all individuals aged 65 years and above in the sampled villages. The sample size was determined by using the formula for single population proportion by considering assumptions like, a 95% confidence level, and 5% margin of error. The largest sample size was selected as shown below (21):

$n={\left(Z_{a/2}\right)}^{2}p\left(1-p\right)/d^{2}$ .

According to the following formula, a total of 1,167 people were included in the study, with 519 men (44.5%) and 648 women (55.5%). Ethics approval was granted by the Committee for Medical Research Ethics of the Center for Disease Control and Prevention in Qingdao (no. spaq-2016-125; date: 03-20-2018).

Inclusion criteria were as follows: age:≥65 years old; Living locally for at least 1 year; volunteer to participate in the survey and sign informed consent; those with normal hearing and speech expression with no communication barriers; able to complete the relevant questionnaires independently or with assistance from relevant personnel.

Exclusion criteria were as follows: patients with severe physical dysfunction; those with cognitive impairment unable to communicate effectively; those with a diagnosis of a definite mental illness; those with severe impaired hearing and vision; those who were unwilling to participate in this study and had bad low compliance; elderly people with incomplete survey data results.

### 2.2 Measurements

#### 2.2.1 General status questionnaire

The elderly individual’s basic information includes gender, age, education, marital status, source of income, and living arrangement.

#### 2.2.2 The Pittsburgh sleep quality index

The study’s outcome variable was the status of sleep quality, assessed using the Pittsburgh Sleep Quality Index. The PSQI scale includes 19 self-assessed and 5 other-assessed items. These items are divided into 7 dimensions: subjective sleep quality, sleep onset latency, sleep duration, sleep efficiency, sleep disturbances, use of sleep medication, and daytime dysfunction. Each dimension is rated from 0 to 3, and the total PSQI score is the sum of these ratings, ranging from 0 to 21. A higher score indicates poorer sleep quality (22). A global PSQI score greater than 5 yielded a diagnostic sensitivity of 89.6% and specificity of 86.5% (kappa = 0.75, *p* less than 0.001) in distinguishing good and poor sleepers (23).

#### 2.2.3 Dietary survey

The study utilized a Food Frequency Questionnaire (FFQ) consisting of 97 food items to assess the dietary intake of participants over the past 3 months based on previous studies (24). The 24-h dietary recall (24-HDR) is a method of assessing an individual’s food intake over the past 24 h using food diaries, models, or household utensils (25). The 24-HDR primarily asks the participants about the types and quantities of food consumed in the previous 24 h, including breakfast, lunch, dinner, snacks, fruits, beverages, supplements, and other items (26). Interviewers may use structured or open-ended questionnaires, sometimes supplemented with visual aids such as pictures, photography examples, and recipe components (27) to collect this information. The 24-HDR typically takes about 25–30 min to complete and does not specify any particular day of the week (28). Nutrient intake was calculated based on a nutritional calculator. These questionnaires have been extensively employed to investigate dietary habits when examining the correlation between diet and disease in rural Chinese populations (29, 30).The cut offs for food and nutrients are determined in accordance with the 2022 Chinese Dietary Guidelines for Residents.

### 2.3 Quality control

The researchers involved in this study were graduate students and senior undergraduates who had the necessary expertise in the field to conduct the survey. The questionnaires were administered in a face-to-face, one-on-one format, and were collected, verified, and entered into Epidata 3.2 for data analysis.

### 2.4 Statistical methods

LCA was conducted using MPlus 8.3 software to assess sleep quality among elderly individuals. Model fitness indicators included Akaike information criterion (AIC), Bayesian information criterion (BIC), Adjusted Bayesian information criterion (aBIC), Lo-mende-l rubin corrected likelihood ratio test (LMRT), Bootstrap likelihood ratio test (BLRT), and entropy. Smaller values of AIC, BIC, and aBIC indicate a better fit, while a *p*-value of <0.05 for LMRT and BLRT suggests a better fit for class k compared to class k-1. An entropy value closer to 1 indicates higher classification accuracy, with values≥0.8 representing a classification accuracy rate of over 90% (11). After determining the best fitting model, SPSS27 software was used to analyze factors influencing sleep quality among elderly individuals in different latent classes using descriptive analysis, chi-square test, one-way ANOVA, and binary logistic regression. Statistical significance was set at *p* < 0.05.

## 3 Results

### 3.1 Basic characteristics of the study subjects

Table 1 depicts the characteristics of the sample population, consisting of 1,167 individuals, of whom 44.5% were male participants. The age was 72.24 ± 5.97 years, with most having received only an elementary school education or below (80.9%). Most were non-smokers (75.1%) and abstained from alcohol (73.2%), with over 72.2% spending more than ￥3,000 on their diet (24).

**Table 1:** Basic characteristics of the elderly.

Characteristics	Frequency	%
Sex		
Man	519	44.5
Woman	648	55.5
Educational status		
Illiterate	527	45.2
Primary school	417	35.7
Middle school and above	223	19.1
Marital status		
Unmarried	16	1.4
Married	883	75.7
Widowed	268	23
Occupation		
Peasant	1,089	93.3
Individual business	30	2.6
Worker	28	2.4
Other	20	1.7
Smoke		
Yes	291	24.9
No	876	75.1
Drink		
Yes	313	26.8
No	854	73.2
Dietary consumption		
≤ 3,000 yuan/month	324	27.8
> 3,000 yuan/month	843	72.2
Income		
≤3,000 yuan/month	219	18.8
>3,000 yuan/month	948	81.2
Family population		
≤2	770	66
﹥2	397	34

### 3.2 Results of the latent class analysis of sleep quality

This study examined different patterns of sleep characteristics in older adults using the PSQI, and the model fit statistics are presented in Table 2. The values of AIC, BIC, and aBIC decreased as the number of classes increased, indicating better fit with more classes. The LMRT *p*-value was <0.001 for 2 classes and 0.127 for 3 classes, which was not statistically significant. The BLRT *p*-value was <0.001 for classes 2 to 5. The Entropy value was >0.8 for classes 2 to 5. The class probabilities decreased as the number of classes increased, with the lowest probability >0.1 for 2 classes and 0.013 for 3 classes. Overall, the model fit was best with 2 classes, suggesting that older adults’ sleep quality can be categorized into 2 distinct classes. Table 3 displays the probabilities of scoring within each category of sleep quality, offering a visual depiction of the chances of an entity achieving a particular score in a specific sleep quality category.

**Table 2:** Latent class model adaptation indicators of sleep characteristics in older adults.

	Log (L)	AIC	BIC	aBIC	LMRT	BLRT	Entropy	Relative frequence of smallest class(%)
1	−8175.941	16379.881	16450.226	16405.759	–	–	–	–
2	−6882.461	13808.921	13919.464	13849.586	0	<0.001	0.993	0.891/0.109
3	−5970.143	12000.287	12151.026	12055.738	0.113	<0.001	0.995	0.013/0.882/0.105
4	−5114.532	10305.064	10,496	10375.302	0.706	<0.001	0.996	0.013/0.871/0.013/0.102
5	−4797.053	9686.105	9917.239	9771.13	0.654	<0.001	0.997	0.013/0.781/0.106/0.086/0.013

Log(L), log likelihood; AIC, Akaike Information Criterion; BIC, Bayesian Information Criterion; aBlC, adjusted Bayesian Information Criterion; LMR-LRT, Lo–Mendell–Rubin Likelihood Ratio Test; BLRT, Bootstrap Likelihood Ratio Test. –, not applicable.

**Table 3:** Probability of scoring each sleep problem category.

PSQI	Class1(0.89057)	Class2(0.10943)
Subjective sleep quality	0.568	1.16
Sleep latency	0.628	1.981
Sleep duration	0.181	2.331
Habitual sleep efficiency	0.115	2.493
Sleep disturbances	0.955	1.146
Daytime functionality	0.313	0.598
Use of sleep medications	0.037	0.145

### 3.3 Latent class characteristics of sleep quality in the elderly

**Figure 1:**
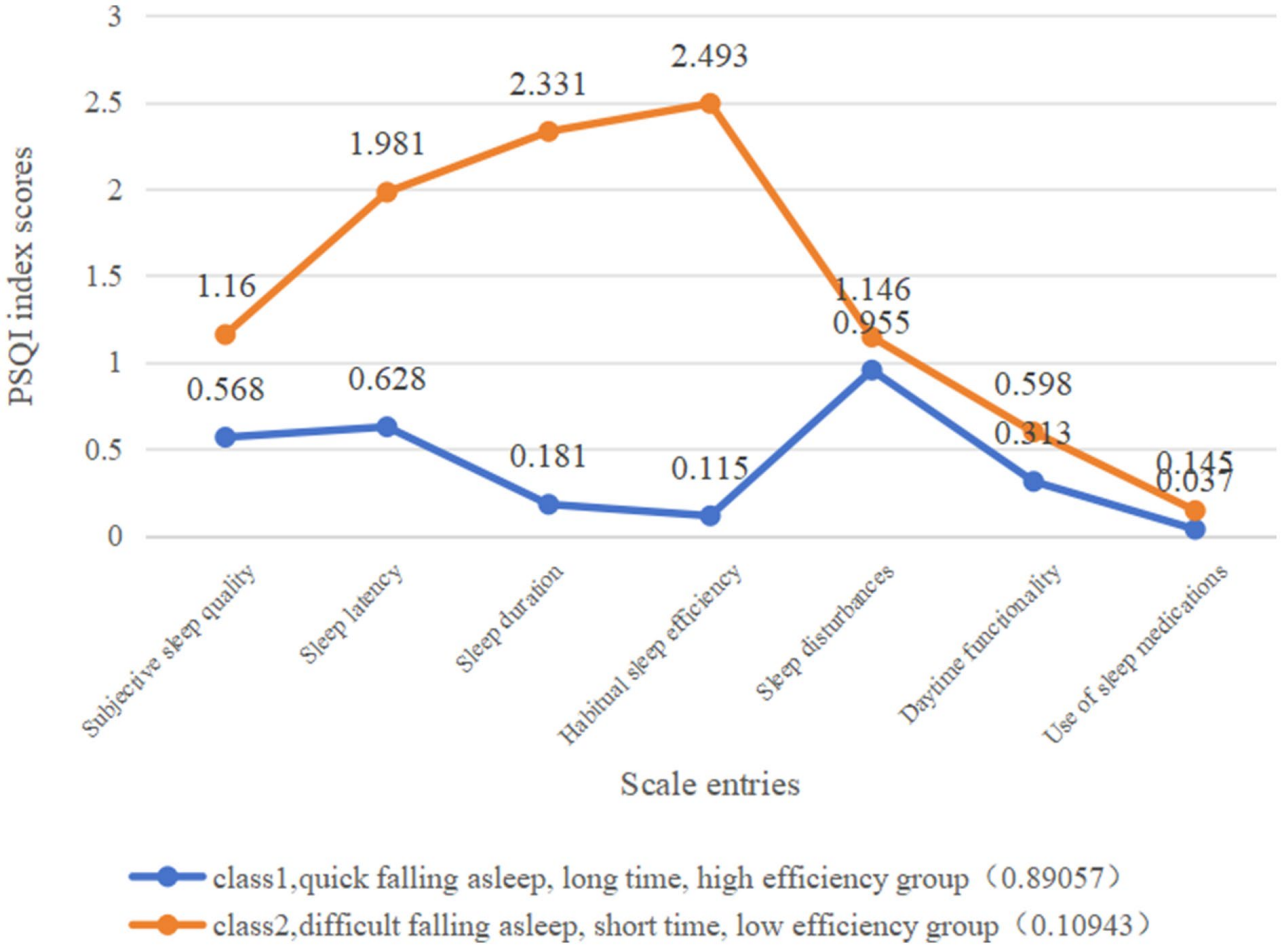
Conditional probability plots of two latent classes of sleep status in rural older adults.

In this study, the sleep quality of rural older adults was classified into two latent classes (Figure 1). Class 1, which comprised 89% of the cases, had lower scores compared to Class 2 in various aspects such as sleep quality, time taken to fall asleep, duration of sleep, sleep efficiency, sleep disorders, daytime functioning, and use of hypnotic medication. The overall sleep score for Class 1 was 2.75, indicating better sleep quality. On the other hand, Class 2, which consisted of 11% of the cases, scored higher than Class 1 in all PSQI indicators, with a total sleep score of 4.96, indice = 0.05. The results indicated differences in the quality of sleep among the elderly subgroups based on their poorer sleep quality. This group was experienced difficulties in falling asleep, had low sleep efficiency, and woke up easily or early at night. Consequently, Class 1 has been labeled as the “quick falling asleep, long time, high efficiency group” (89.1%), while Class 2 has been denoted as the “difficult falling asleep, short time, low efficiency group” (10.9%).”

### 3.4 Food and nutrient intake for different sleep classes

The study examined the sleep patterns of older adults based on their food and nutrient intake. Using a Chi-square test with a significance level of *α* = 0.05, the analysis found differences in sleep quality among older adults in various subgroups. The results revealed that the sleep quality of older adults in different subgroups differed in terms of their daily food intake of rice, noodles, meat, potatoes, dairy, and soybeans, and daily nutrient intake of proteins, lipids, retinol, niacin, vitamin C, vitamin E, magnesium, potassium, sodium, and iron. Details are shown in Tables 4, 5.

**Table 4:** Univariate analysis of food for different sleep typologies in elderly people.

Variable	Class1	Class2	X^2^	*p* value
Rice and noodles intake				
<200 g/d	177(84.3)	824(90.2)	6.032	0.017
≥200 g/d	33(18.7)	90(9.8)		
Potato intake				
<50 g/d	698(87.8)	303(92.1)	4.412	0.036
≥50 g/d	97(12.2)	26(7.9)		
Whole grains and mixed beans intake				
<50 g/d	464(46.4)	61(57.5)	0.462	0.497
≥50 g/d	537(53.6)	62(65.5)		
Coarse grains intake				
<200 g/d	544(54.3)	67(66.9)	0.001	0.979
≥200 g/d	457(45.7)	56(56.1)		
Soybeans intake				
<50 g/d	808(88)	193(93.7)	5.554	0.023
≥50 g/d	110(12)	13(6.3)		
Nut intake				
<10 g/d	706(70.5)	95(77.9)	2.864	0.091
≥10 g/d	295(29.5)	27(22.1)		
Livestock and poultry meat intake				
<40 g/d	374(86)	627(91)	6.907	0.009
≥40 g/d	61(14)	62(9)		
Fish, shrimp and crabs intake				
<45 g/d	863(86.2)	101(82.1)	1.508	0.219
≥45 g/d	138(13.8)	22(17.9)		
Vegetables intake				
<300 g/d	378(37.8)	51(42.3)	0.945	0.331
≥300 g/d	623(62.2)	72(57.7)		
Fruit intake				
<200 g/d	637(63.6)	82(78.7)	0.436	0.509
≥200 g/d	364(36.4)	41(44.3)		
Milk intake				
<300 g/d	983(89.4)	11(72)	8.135	0.004
≥300 g/d	116(10.6)	6(28)		

**Table 5:** Univariate analysis of nutrients for different sleep classes in the elderly.

Variable	Class1	Class2	X^2^	*p* value
Carbohydrate intake				
<250 g/d	476(47.6)	61(49.6)	0.183	0.669
≥250 g/d	525(52.4)	62(50.4)		
Protein intake				
<50 g/d	192(85.3)	809(90)	4.002	0.045
≥50 g/d	33(14.7)	90(10)		
Lipids intake				
<25 g/d	34(3.4)	12(9.8)	11.287	<0.001
≥25 g/d	967(96.6)	111(90.2)		
Retinol intake				
<700 μg RE/d	966(89.5)	35(77.8)	4.973	0.026
≥700 μg RE/d	113(10.5)	10(22.2)		
Thiamine intake				
<1 mg/d	572(57.1)	75(61.0)	0.659	0.417
≥1 mg/d	429(42.9)	48(39.0)		
Riboflavin intake				
<2 mg/d	981(98.0)	117(95.1)	2.847	0.092
≥2 mg/d	20(2.0)	6(4.9)		
Niacin intake				
<10 mg/d	250(25.0)	50(40.7)	13.756	<0.001
≥10 mg/d	751(75.0)	73(59.3)		
Vitamin C intake				
<100 mg/d	632(63.1)	88(71.5)	3.363	0.067
≥100 mg/d	369(36.9)	35(28.5)		
Vitamin E intake				
<15 mg/d	21(72.4)	980(89.5)	6.799	0.009
≥15 mg/d	8(27.6)	115(10.5)		
Magnesium intake				
<300 mg/d	264(85.4)	737(90.4)	5.730	0.017
≥300 mg/d	45(14.6)	78(9.6)		
Potassium				
<2,000 mg/d	385(38.5)	63(51.2)	7.438	0.006
≥2000 mg/d	616(61.5)	60(48.8)		
Sodium intake				
<1,200 mg/d	186(18.6)	37(30.1)	9.109	0.003
≥1,200 mg/d	814(81.4)	86(69.9)		
Calcium intake				
<800 mg/d	916(91.5)	110(89.4)	0.594	0.441
≥ 800 mg/d	85(8.5)	13(10.6)		
Iron intake				
<15 mg/d	181(18.6)	32(26.0)	3.873	0.049
≥15 mg/d	815(81.4)	91(74.0)		
Mn intake				
<2.5 mg/d	34(3.4)	5(4.1)	0.146	0.702
≥2.5 mg/d	967(96.6)	118(95.9)		
Zinc intake				
<12 mg/d	411(41.1)	60(48.8)	2.683	0.101
≥12 mg/d	590(58.9)	63(51.2)		
Copper intake				
<2 mg/d	534(53.3)	74(60.2)	2.049	0.152
≥2 mg/d	467(46.7)	49(39.8)		
Phosphate intake				
<750 mg/d	161(16.1)	27(22.0)	2.707	0.100
≥750 mg/d	840(83.9)	96(78.0)		
Se intake				
<50 ug/d	372(37.2)	47(38.2)	0.052	0.820
≥50 ug/d	629(62.8)	76(61.8)		

### 3.5 Binary logistic regression analysis for two classes of sleep quality

A binary logistic regression analysis was conducted using the sleep quality classification obtained from the latent class analysis as the dependent variable. The class 1 was used as the reference group, and the significant variables from the univariate analysis were utilized as independent variables.

The analysis revealed that in terms of dietary nutrition, compared to the class 1, older adults in the class 2 were more likely to have a daily intake of potatoes <50 g (OR = 1.863, *p* = 0.034) and lipids ≥25 g (OR = 3.73, *p* = 0.007) as risk factors for sleep quality. Conversely, those in the class 1 were more likely to have a daily intake of protein ≥60 g (OR = 0.367, *p* = 0.006), retinol ≥700μgRE equivalents (OR = 0.212, *p* = 0.002), and dairy ≥300 g (OR = 0.295, *p* = 0.035) as protective factors for sleep quality.

## 4 Discussion

This study investigated the relationship between dietary nutrients and sleep quality in rural elderly individuals by categorizing latent classes of sleep characteristics using the PSQI. The findings revealed that 12.3% of the participants (*n* = 143) experienced sleep disorders, indicating a notable prevalence of sleep issues in this demographic. Specifically, participants reported longer sleep onset latency and increased sleep disturbances. In comparison, a meta-analysis of elderly Chinese individuals reported a higher prevalence rate of 41.2% (31). Discrepancies in detection rates may be attributed to variations in sampling methods, survey tools, sample sizes, and participant selection criteria. Model testing indicated that a two-class solution best fit the data, identifying two latent classes among rural elderly individuals: "class 1 (89.1%)” and the “class 2 (10.9%).” LCA was employed to explore individual variations in latent classes rather than their levels, focusing on categorical data analysis and shared trait probabilities. Most research evaluates the sleep quality of elderly individuals using the PSQI index sorely (10). This study was the first to employ LCA for classifying sleep quality in this population, providing a more suitable approach for examining the relationship between dietary nutrients and diverse sleep patterns (Table 6).

**Table 6:** Dichotomous Logistic regression analysis of factors influencing different sleep classification in older adults.

Variable	B	SE	Wald	Ex P (B)	95% CI	*p* value
Potatoes (Take ≥50 g/d as a reference)						
<50 g/d	0.622	0.294	4.478	1.863	1.047–3.315	0.034
milk (Take <300 g/d as a reference)						
≥300 g/d	−1.222	0.58	4.445	0.295	0.095–0.095	0.035
protein (Take <60 g/d as a reference)						
≥60 g/d	−1.001	0.362	7.651	0.367	0.181–0.747	0.006
lipid (Take <25 g/d as a reference)						
≥25 g/d	1.316	0.487	7.300	3.730	1.435–9.693	0.007
Retinol equivalents (Take <700μgRE as a reference)						
≥700μgRE	−1.551	0.504	9.448	0.212	0.079–0.57	0.002

Older adults in class 2 exhibited a higher tendency to consume fewer potatoes and higher amounts of lipids daily, identified as risk factors, while displaying a reduced likelihood of consuming more protein, retinol, and dairy daily, recognized as protective factors for sleep quality compared to class 1. The results suggest that older individuals consuming over 50 g of potatoes daily may experience quicker sleep onset, prolonged sleep duration, and improved sleep efficiency. Prior to this, studies on sleep quality did not consider the intake of potatoes. This is due to the high starch content in potatoes, which contains tryptophan that aids in serotonin production, a neurotransmitter that can be transformed into melatonin (32). Melatonin plays a crucial role in regulating sleep–wake patterns, and a lack of tryptophan can lead to sleep disturbances (33).Melatonin plays a crucial role in regulating the immune system by enhancing lymphocyte activity and cytokine production. It also helps in regulating energy metabolism, delaying aging, and improving the quality of life in old age. Studies have shown that polyphenols found in potatoes and tea can promote the growth of beneficial gut bacteria, leading to the production of anti-inflammatory compounds that support the immune system and improve sleep (34). Therefore, this study suggests that older adults in rural settings should consider consuming more than 50 grams of potatoes daily to potentially enhance sleep efficiency and duration.

Moreover, that elderly individuals who consumed at least 300 g of milk daily had better sleep quality, falling asleep faster, sleeping longer, and being more efficient. This suggests that adequate milk intake is a protective factor for sleep quality, supported by previous research (35). Yet, there is currently no research that has delved into the relationship between daily milk intake and sleep quality. It has been recommended to consume milk before bedtime, as it is rich in tryptophan which is essential for melatonin synthesis and acts as an antioxidant. Additionally, supplementation with tryptophan-fortified and fermented dairy products can help improve sleep disorders (36). The Dietary Guidelines for Chinese Residents 2022 recommend daily milk consumption (300–500 g) for elderly individuals to improve sleep quality.

This study conducted on rural older adults revealed that consuming a diet high in protein and low in fat is linked to better sleep quality. This finding aligns with the results of the current study. Increasing protein intake has been shown to have a positive impact on sleep quality (37) as it was associated with longer sleep duration and improved sleep efficiency. Typically, a variety of foods are known to be protein-rich, with common high-quality sources such as milk, eggs, meat, and beans, which have been associated with improved sleep quality. Adequate protein intake can support the repair and regeneration of various body parts, contributing to better sleep quality. Protein-rich foods contain tryptophan, a key precursor for melatonin synthesis. Melatonin is a hormone that regulates sleep, helping to reduce the time it takes to fall asleep and wake up, alleviate sleep disorders, decrease the number of awakenings during sleep, and enhance the duration of deep sleep while reducing light sleep periods. This overall has a positive impact on sleep quality. In this study, it was suggested that rural elderly individuals who consume at least 60 g of protein per day are more inclined to belong to the class 1. As a result, it is recommended that rural elderly individuals with a daily protein intake of 60 g or more work toward improving their sleep quality.

Moreover, consuming protein can help regulate blood sugar levels, leading to better sleep quality for elderly individuals living in rural area (38). Stable blood sugar levels make it easier for the body to enter deep sleep and reduce the chances of waking up during the night, resulting in longer and more restful sleep. High-protein foods are more effective than high-sugar foods in maintaining stable blood sugar levels. It is important to note, however, that excessive protein intake can strain the kidneys and potentially cause other health issues, especially as liver and kidney function naturally decline with age. Hence, it is advisable to adhere to a balanced diet, ingest moderate quantities of protein (≥ 60 g/d), and prioritize high-quality protein sources like milk, eggs, meat, beans, among others, to guarantee adequate nutrition while mitigating health risks (39).

This study indicates that an elevated lipid intake may adversely affect sleep quality, particularly among elderly individuals residing in rural areas. Limited research has been conducted on the correlation between macronutrient intake and sleep quality. In essence, the consumption of different types and quantities of carbohydrates and fats has shown associations with varying sleep quality outcomes. Higher protein diets, on the other hand, have been linked to improved sleep quality (19). This study indicates that individuals consuming 25 g or more of lipids daily have a higher probability of being classified into the class 2. To enhance sleep quality among elderly individuals in rural regions, it is advisable to restrict lipid intake to less than 25 g per day, steer clear of high-fat foods, adhere to a well-rounded diet, and engage in regular physical activity to manage weight and enhance sleep quality.

This study found that elderly individuals who consume more than 700μgRE of retinol equivalents daily tend to have better sleep quality. Previous research often only focused on the impact of retinol on sleep quality, without analyzing its relationship with sleep characteristics. Retinol activity equivalents are a good indicator of vitamin A levels in the body. Foods rich in vitamin A mainly include fruits, vegetables, meat, legumes, and animal organs. Vitamin A is crucial for brain function, as research has shown that a deficiency in vitamin A can disrupt circadian rhythms, leading to circadian rhythm disturbances and sleep disorders (40). Adequate intake of vitamin A in elderly individuals has been linked to better sleep quality (41). Numerous studies have shown that consuming vitamin A can improve sleep quality. Vitamin A may indirectly regulate sleep by interacting with the neurotransmitters dopamine and acetylcholine (42) or it could be due to its ability to boost the body’s immune response and resistance to infections, thereby promoting overall health and enhancing sleep.

The cross-sectional study was subject to several limitations. Firstly, the study was predominantly observational or cross-sectional in nature, hinders the ability to track changes in sleep quality among rural older adults and establish causal relationships between dietary nutrient intake and sleep patterns in this demographic. Secondly, the study’s restriction to rural areas in Shandong province may impede the generalizability of the findings. Thirdly, the reliance on self-reported measures can introduce recall bias, potentially impacting the results of the latent class analysis. Therefore, additional high-quality cohort studies and randomized controlled trials (RCTs) are necessary to advance knowledge of the connection between dietary nutrient intake and sleep patterns in rural older adults.

## 5 Conclusion

Our research categorizes the sleep patterns of elderly individuals into two groups: “Quick falling asleep, long time, high efficiency group” (89.1%) and “difficult falling asleep, short time, low efficiency group” (10.9%). Our findings indicate that improving the intake of dairy products (300-500 g/day), potatoes (≥ 50 g/day), foods rich in retinol (≥ 700 μg RE), and protein (≥ 60 g/day), while reducing the consumption of high-fat foods (≤ 25 g/day) can potentially enhance the sleep quality of elderly individuals residing in rural areas.

## Data Availability

The raw data supporting the conclusions of this article will be made available by the authors, without undue reservation.
